# The last resort: Workplace bullying and the consequences of changing jobs

**DOI:** 10.1111/sjop.12794

**Published:** 2022-01-21

**Authors:** Michael Rosander, Denise Salin, Stefan Blomberg

**Affiliations:** ^1^ Department of Behavioural Sciences and Learning Linköping University Linköping Sweden; ^2^ Department of Management and Organisation Hanken School of Economics Helsinki Finland; ^3^ Department of Health, Occupational and Environmental Medicine Center, Medicine and Caring Sciences Linköping University Linköping Sweden

**Keywords:** Workplace bullying, job change, mental health problems, anxiety, depression, employee turnover

## Abstract

This study investigated the consequences of changing jobs for employees subjected to workplace bullying. First, we hypothesized that bullied employees would be more likely to change jobs than non‐bullied employees. Moreover, we hypothesized that changing jobs would result in a reduction of exposure to bullying behaviors and an alleviation of mental health problems for those bullied at baseline. The study was based on a longitudinal probability sample of the whole Swedish workforce (*n* = 1,095). The time lag was 18 months. The results supported all hypotheses except one. Those employees who were bullied at baseline were more likely to have changed jobs at follow‐up. Also, for the changers there was a reduction in exposure to subsequent bullying. The actual drop in exposure to bullying behaviors was significant and substantial. This gives further support for the work environment hypothesis, suggesting the work context may be a more important cause than individual characteristics. As for mental health problems, the association between bullying and subsequent anxiety was not significant for those changing jobs, suggesting that leaving a toxic workplace may reduce anxiety relatively quickly. However, depression symptoms were not affected by the change of jobs, and the association between bullying and subsequent depression was the same 18 months later. The conclusion is that changing jobs can be a useful, last resort on an individual level, improving the situation for the victim of bullying. However, it is important to note that it does not solve any underlying organizational problems and risk factors.

## INTRODUCTION

Workplace bullying is defined as systematic exposure to negative behaviors at work over an extended period of time in situations where the exposed have little or no possibilities to defend themselves (Einarsen, Hoel, Zapf & Cooper, 2020). There are many studies connecting exposure to bullying to subsequent mental health problems, for example, depression and anxiety (e.g., Bonde, Gullander, Hansen *et al*., 2016; Einarsen & Nielsen, 2015; Gullander, Hogh, Hansen *et al*., 2014; McTernan, Dollard & LaMontagne, 2013; Reknes, Pallesen, Mageroy, Moen, Bjorvatn & Einarsen, 2014), and sleep problems (e.g., Hansen, Hogh, Garde & Persson, 2014; Nielsen, Pallesen, Einarsen, Harris, Rajalingam & Gjerstad, 2021; Nielsen, Harris, Pallesen & Einarsen, 2020). In fact, in a five‐year longitudinal study Nielsen, Nielsen, Notelaers, and Einarsen (2015) showed that the risk of suicide ideation doubled if exposed to workplace bullying. Furthermore, bullying has been shown to increase the risk of long‐term sickness (e.g., Grynderup, Nabe‐Nielsen, Lange *et al*., 2017) and unemployment (e.g., Glambek, Skogstad & Einarsen, 2015). Given such potentially serious consequences, victims of bullying may feel they have no choice but to get away as a survival tactic – in other words, to change jobs before it is too late. Despite this, so far we know little about what happens when victims of bullying actually change jobs. Therefore, this study aims to investigate whether changing jobs results in improved outcomes for victims.

Previous research has examined intention to leave and turnover as consequences of workplace bullying, and found support that bullying increases such tendencies (e.g., Nielsen, Grynderup, Conway *et al*., 2017; Reknes, Glambek & Einarsen, 2020). However, studies investigating the actual effects of changing jobs are scarce. Brousse, Fontana, Ouchchane *et al*. (2008) investigated mental health problems of patients referred to a clinic for treatment, of whom some had changed jobs. However, it was a small study and only ten patients had changed jobs at follow‐up. As such, job changers were not analysed separately. Similarly, Schwickerath (2009, cited in Schwickerath & Zapf, 2020) investigated the effects of psychotherapy for bullying victims and of these victims some had changed jobs after therapy. However, again the job change as such was not the focus of the study. It thus remains unclear whether changing jobs actually results in a reduction of bullying behaviors or not. To the extent that bullying is caused by factors in the work environment (as suggested by the work environment hypothesis, cf. Einarsen, 2000; Leymann, 1996; Salin & Hoel, 2020), a change of jobs could be expected to result in the bullying ending, as a change of jobs would also result in a changed work environment. However, to the extent that bullying is the result of individual factors, such as personality traits of the victim, changing jobs may provide little relief. Examining whether changing jobs actually results in a reduced exposure to bullying behaviors may thus also indirectly provide us with insights into the causes of bullying.

As discussed above and as evidenced by a wealth of both cross‐sectional and longitudinal studies, bullying has severe negative consequences for the health and well‐being of those exposed, and the effects can be long‐lasting (Boudrias, Trépanier & Salin, 2021; Nielsen & Einarsen, 2012). However, again, we know little about how a change of jobs affects this. Will changing jobs end the distress experienced by those exposed to bullying, or will the negative consequences linger despite such a change?

The aim of this study is to provide new knowledge on the consequences of changing jobs for an individual who is exposed to workplace bullying. By providing insights on the consequences of changing jobs, the study also indirectly increases understanding of the role of external vs. individual risk factors of bullying. Similarly, it increases our understanding of the persistence of health consequences of bullying. The research questions are explored using a probability sample drawn from the total pool of the Swedish working population, consisting of a total of 1,095 respondents, including 182 respondents who were classified as being exposed to at least occasional bullying.

## BULLYING, JOB CHANGE AND CONSEQUENCES

### Intention to leave and actual turnover

Turnover is typically a way to describe normal cycles in organizations – people leave and are replaced. The people who leave can end up unemployed or on disability retirement, but in many cases voluntary turnover simply means that a person is leaving for a new job. Early studies focused mainly on job satisfaction and organizational commitment as antecedents of turnover (Tett & Meyer, 1995). As an alternative to the traditional models of turnover, Lee and Mitchell (1994) suggested an unfolding model of voluntary turnover starting with a shock to the system. Lee and Mitchell (1994, p. 1993) described this as “a very distinguishable event that *jars* employees toward deliberate judgments about their jobs.” Although framing it as a shock, the event does not need to come as a surprise to the employee – it may merely be something that “shakes an employee out of a steady state” Lee and Mitchell (1994), p. 1994), that makes one reconsider or think about one's job. Shocks can be positive such as a surprising and exciting job offer, or negative such as a clash with a co‐worker (Holtom, Mitchell, Lee & Inderrieden, 2005). Shocks can also be neutral, but no matter the direction, the event is evaluated using the organization as a context. As a second process, according to the model, the employee evaluates whether the event can be dealt with or responded to with ease. Systematic mistreatment at work may come to a point at which the mistreated employee starts considering alternatives to the current job. Prolonged negative treatment may lead to feelings of resignation with little or no real hope of stopping the bullying oneself (Nielsen & Einarsen, 2018). Based on the unfolding model of voluntary turnover, it is reasonable to assume that the realization that one is a victim of bullying increases the risk or chance of leaving one's job.

Research on bullying and turnover or turnover intentions supports the assumed association between bullying and leaving one's job. In a random sample drawn from the Norwegian workforce, Glambek *et al*. (2015) showed that those who were bullied had a doubled risk of changing jobs over a five‐year period. Other studies focusing on actual turnover have examined specific types of organizations or sectors, but have produced similar results. Investigating self‐labeled bullying among care workers in the Danish eldercare services, Clausen, Hogh, Carneiro, and Borg (2013) found an increased risk of job change: an odds ratio of 1.8 or 2.7, depending on the severity of exposure. Furthermore, Clausen, Hansen, Hogh *et al*. (2016) studied three occupational groups: human service and sales workers, office workers, and manual workers. However, they found an increased risk only for office workers (the risk was about doubled). Finally, Nabe‐Nielsen *et al*. (2017) found a small but significant increase in risk for turnover among civil servants from a large Danish cohort (OR = 1.35) as a result of bullying. It is interesting to note that all but Glambek *et al*. (2015) used self‐labeled bullying as the measure of bullying. Comparisons of different measurement methods show that choice of measurement affect who is classified as a target of bullying, possibly affecting results (Nielsen, Notelaers & Einarsen, 2020; Rosander, Salin, Viita & Blomberg, 2020). Also, the data used in the above‐mentioned four studies are more than ten years old, collected between 2005 and 2010. Thus, trying to replicate the findings in a recent sample may be of relevance.

In addition, there are a few studies investigating the association between workplace bullying and intention to leave (e.g., Glambek, Matthiesen, Hetland & Einarsen, 2014; Reknes *et al*., 2020; Trepanier, Fernet & Austin, 2015). The intention to leave one's current employment is an important predictor of actual turnover (Tett & Meyer, 1995), although not everyone wanting to leave their job may have the resources or possibilities to do so. Also, actually leaving one's job does not always result in finding a new one. Glambek *et al*. (2015) found a more than fourfold risk for those who are bullied to become unemployed, and an almost threefold risk for disability retirement. For disability retirement, similar results have been presented by Nielsen, Emberland, and Knardahl (2017). Exposure to workplace bullying has also been linked to an increased risk of dropout from one's profession (Hogh, Giver, Hannerz & Pedersen, 2012).

Based on the unfolding model of voluntary turnover (Lee & Mitchell, 1994) and the previous studies focusing on turnover (Clausen *et al*., 2013; Clausen *et al*., 2016; Glambek *et al*., 2015; Nabe‐Nielsen *et al*., 2017) we expect to find the following:

Hypothesis 1 Employees who are bullied show a greater tendency to change jobs. In other words, there is a greater risk for employees who were bullied at baseline to have changed jobs at follow‐up compared to the non‐bullied.

### Changing jobs and the risk of workplace bullying

When looking at what causes bullying to occur, a large body of research points to conditions in the work environment as the main cause (Salin & Hoel, 2020). Main risk factors include, for example, role conflict and role ambiguity (Bowling & Beehr, 2006; Van den Brande, Baillien, De Witte, Vander Elst & Godderis, 2016). Poor or absent leadership could elevate the risks associated with a disorderly organization (Ågotnes, Einarsen, Hetland & Skogstad, 2018; Salin, 2003). There is a strong empirical support for the impact of the work environment on the risk of bullying – a strong association between workplace bullying and factors such as role ambiguity, role conflict, job demands, laissez‐faire leadership, and lack of clear goals (Baillien, De Cuyper & De Witte, 2011; Reknes, Einarsen, Knardahl & Lau, 2014; Skogstad, Torsheim, Einarsen & Hauge, 2011). Deficits in the work environment leading to contradictory and unclear expectations increase the risk of frustration, conflict, and aggression that could escalate into bullying. There are also studies pointing to individual factors playing a part in the development of bullying. In a meta‐analysis of targets' personality characteristics, Nielsen, Glasø, and Einarsen (2017) showed a small, but significant, association with workplace bullying. They found geographical differences, but in a European context neuroticism showed the strongest association, and a stronger association if self‐labeling as a victim. However, few studies have investigated the longitudinal and cross‐lagged relationship between personality characteristics and workplace bullying to illuminate the causal directions (Nielsen & Einarsen, 2018). Nielsen and Knardahl (2015) concluded that the impact of personality is limited in regard to subsequent exposure to bullying. Studies have also shown an association between mental health problems at baseline and exposure to bullying at follow‐up (Nielsen & Einarsen, 2012). One could argue that the association between individual causes and bullying may depend on the work environment in such a way that individual characteristics may only be associated with subsequent bullying in organization lacking in role clarity or leadership. For example, in a recent study, Rosander (2021) showed that the association between mental health problems, a known individual risk factor for bullying, and subsequent bullying, only was present if there were also organizational deficits present. Regardless of cause, workplace bullying is a long‐lasting phenomenon. On average, based on 18 studies, the duration was more than two years and 8 months (Zapf, Escartín, Scheppa‐Lahyani, Einarsen, Hoel & Vartia, 2020). Workplace bullying has been described as an escalating process (Einarsen *et al*., 2020; Rosander & Blomberg, 2019) and the association between bullying at different points in time is likely to be strong.

Taken together, based on the work environment hypothesis (Einarsen, 2000; Leymann, 1996; Salin & Hoel, 2020), we expect to find less exposure to workplace bullying among those who changed jobs and a weaker association between the level of bullying at baseline and follow‐up.

Hypothesis 2 Changing jobs reduces the risk of subsequent bullying. In other words, changing jobs moderates the association between workplace bullying at baseline and follow‐up showing a weaker association for employees who changed jobs.

### Health consequences of workplace bullying

There are many studies investigating the consequences of workplace bullying (Boudrias *et al*., 2021; Nielsen & Einarsen, 2012). Since the previous large review on outcomes of bullying (Nielsen & Einarsen, 2012), Boudrias *et al*. (2021) found over 50 longitudinal studies of consequences, and over 20 of these had a focus on psychological health outcomes such as depression and anxiety. In a meta‐analysis, Nielsen, Mageroy, Gjerstad, and Einarsen (2014) found consistent support for an elevated risk of subsequent mental health problems as a consequence of exposure to bullying (OR = 1.68). Thus, there seems to be agreement that bullying can have detrimental effects on a person's mental health. However, we still have limited understanding of the mechanisms and as such also limited understanding of the persistence of these consequences.

There are a number of theoretical starting points that could be used to understand the connection between workplace bullying and mental health problems, such as the transactional theory of stress and coping (Lazarus & Folkman, 1984), the cognitive trauma theory (Janoff‐Bulman, 1989), and the cognitive activation theory of stress (CATS; Ursin & Eriksen, 2004). According to CATS a stress response is a normal reaction increasing arousal to help deal with the situation. The way one deals with a stressful situation depends on the acquired expectancy of the outcome. In many cases there is a sense of control and the expectation of a response is positive leading to a reduction in arousal. However, in the case of bullying a more probable expectation would be that any response, trying to retain some control of the situation, will lead to a negative outcome, hopelessness. If the exposure to bullying continues a likely outcome is helplessness – a perception of no control and that there is nothing one can do to reduce the stress. Both hopelessness and helplessness lead to negative health consequences due to sustained arousal (Ursin & Eriksen, 2004). As an extreme stressor, workplace bullying may also have an effect on a person's basic assumptions of the world. According to Janoff‐Bulman's (1989) schema theory of shattered assumptions, traumatic treatment such as prolonged exposure to bullying behaviors may threaten one's fundamental beliefs about a benevolent and meaningful world, and about one's self‐worth. As a consequence this could lead to extreme emotional reactions including fear, helplessness, anxiety, and depression (Mikkelsen & Einarsen, 2002). Removing the immediate stressor, as a change of jobs could achieve, could help start rebuilding trust. In the transactional stress theory there is a focus on appraisal and coping processes in relation to the environment and one's well‐being (Lazarus, 1999). Appraisal processes involve evaluating what is happening, if it is harmful, and what one can do to cope with the situation if it is deemed to be harmful. Being exposed to workplace bullying is an extreme stressor that is characterized by lack of personal resources and loss of control (Zapf & Einarsen, 2005). In an ongoing bullying situation, appraisal may become secondary to coping processes or attempts to cope with the situation. Previous research has shown that coping and personal resources only have an effect on one's mental health when exposure to negative behaviors is relatively low (Nielsen & Einarsen, 2018). When the bullying process escalates, and exposure gets higher, personal resources, such as coping style, agreeableness, and optimism, do not help. In terms of the appraisal theory (Lazarus, 1999), bullying as an extreme stressor will likely lead to an extreme stress reaction. The stress reaction in terms of mental health problems may also fuel continued exposure to bullying. For example, anxiety and fear of facing one's tormentors could make the exposed more cautious and sensitive to future treatment (de Lange, Taris, Kompier, Houtman & Bongers, 2005), but also less capable of carrying out one's duties, possibly creating frustration from co‐workers and continued mistreatment (Zapf & Einarsen, 2020). Aggression can also be triggered by perceived norm violations such as failure to live up to an expected social exchange at work based on a social interactionist perspective (Felson, 1992). If changing jobs, the main focus may be redirected to appraisal processes (Lazarus, 1999). In case the work environment proves less threatening at the new workplace, the responses may be normalized and with that a reduction in possible causes for mental health problems.

Previous research has shown strong support for the association between exposure to bullying and subsequent mental health problems. In terms of the transactional theory of stress and coping (Lazarus & Folkman, 1984), the cognitive trauma theory (Janoff‐Bulman, 1989), and the cognitive activation theory of stress (Ursin & Eriksen, 2004), the severe reactions can be understood. The current study investigates what happens when the immediate stressors are removed when leaving one's job for a new one. Based on the reasons put forward earlier, it is reasonable to assume that this change may have an effect on a person's mental health.

Hypothesis 3 Changing jobs reduces mental health problems, both symptoms of (a) depression, and (b) anxiety, experienced by employees who have been exposed to bullying. In other words, changing jobs moderates the association between workplace bullying at baseline and mental health problems at follow‐up.

## METHODS

### Design and sample

The study used a probability sample drawn from the complete workforce in Sweden working at workplaces with ten or more employees (3.3 m people). The government agency Statistics Sweden (scb.se/en) handled this procedure. Baseline data (T1) were collected in the autumn of 2017 (*n* = 1,854) and the follow‐up data (T2) were collected 18 months later, in the spring of 2019. A total of 1,095 employees responded at both T1 and T2, which is the data used in this study. There were 58% women in the sample. The mean age was 49.3 years (*SD* = 10.0), and the mean period of employment at the current workplace was 13.5 years (*SD* = 11.6). The mean yearly income was SEK 429,000 (*SD* = 207,000). Most had a permanent contract (95%), and 14% had some kind of managerial position. Education was measured in eight levels in terms of length of education: 1% had less than 9 years, 4% had only 9–10 years (compulsory school), and 35% had 11–12 years. The majority had some form of university or college education: 8% had 1 year, 11% had 2 years, 21% had 3 years, 18% had 4–5 years, and 2% had a PhD education or the equivalent. The respondents had on average 0.9 children (*SD* = 1.0) and 54% were married.

The focus of the study is the employees who changed jobs between T1 and T2, the “Changers.” Close to 16% or 174 employees in the sample changed jobs. The ones who did not change jobs will be referred to as “Stayers.” Table 1 presents demographic information about the two groups.

**Table 1:** Differences between Stayers and Changers

	Stayers	Changers	Statistics
Sex (% women)	58%	59%	*ns*
Education (% university)	63%	50%	χ^2^(2) = 11.2, *p* = 0.004
Managerial position (%)	14%	18%	*ns*
Permanent contract (%)	97%	90%	χ^2^(1) = 15.8, *p* < 0.001
Age (years)	50.0 (9.8)	45.1 (10.5)	*t* = 6.1, *p* < 0.001
Period of employment (years)	14.3 (11.8)	9.7 (9.7)	*t* = 4.8, *p* < 0.001
Income	4.27 (1.98)	4.44 (2.52)	*ns*

*Note*: Mean and standard deviation in parenthesis for continuous variables. Income = yearly income in hundred thousand SEK.

As for differences between the two groups it can be noted that there were significantly fewer with a university education among the Changers, and fewer with a fixed contract (however, a very large majority in both groups had a fixed contract). The Changers were also significantly younger and had worked a shorter time in the organization that they left.

### Measures

Whether a respondent had changed jobs or not since T1 was measured using a direct yes/no question: “Have you changed jobs since the last survey (i.e., since the autumn of 2017)?” In the study we also measured workplace bullying and mental health. For bullying we used the Negative Acts Questionnaire–Revised (NAQ–R, Einarsen, Hoel & Notelaers, 2009; Rosander & Blomberg, 2019), as well as a single item for self‐labeling as bullied based on a definition. The NAQ–R and the self‐labeling item both have the same five‐point frequency scale: never, now and then, monthly, weekly, and daily. The NAQ–R consists of 22 items covering a range of negative acts one can be exposed to at work. Examples of items are “Being ignored or excluded” and “Persistent criticism of your errors or mistakes.” Cronbach's alpha for the NAQ–R was 0.89 at T1, and 0.90 at T2. In addition to sum and mean scores, we used cut‐off scores. For the NAQ–R, Notelaers and Einarsen (2013) suggested a sum of 33 or higher to represent “occasional bullying” and a sum of 45 or higher for “victim of bullying.” The cut‐off score for self‐labeled bullying was at least “now and then.”

We used the Hospital Anxiety and Depression Scale (HADS, Zigmond & Snaith, 1983) to measure mental health. The HADS consists of 14 items on a scale from 0 to 3. Cronbach's alpha for the HADS as a whole was 0.90 at T1 and 0.89 at T2. In the analyses for H3 we separated the items measuring anxiety (HADS–A, 7 items) and depression (HADS–D, 7 items). Cronbach's alpha for HADS–A was at T1 0.85 and at T2 0.84, and for HADS–D at T1 0.84 and at T2 0.84. A sample item for depression symptoms is “I feel cheerful,” and for anxiety symptoms “I feel tense or wound up” with possible responses from “Not at all” to “Most of the time.”

### Control variables

There are many reasons for a person to change jobs, and there are some factors that may make the change harder to go through with (Rubenstein, Eberly, Lee & Mitchell, 2018). In terms of predicting a change of jobs based on exposure to bullying, having less opportunities to change may be a confounding factor. A person may wish to change jobs to get away from bullies, but does not feel he or she can do it because of problems getting a new job – perhaps based on having less education. A lower education may provide less opportunities. Having a higher income could also be an indication that a person may find it easier to change jobs. Although one's education says something about one's income (*r* = 0.26, *p* < 0.001), a high education is not a guarantee for a good income, so it is reasonable to include both. Age and income are also positively related (*r* = 0.19, *p* < 0.001), but age may also have a reversed effect on opportunities to change jobs in that young people feel freer to change as there are fewer obligations, such as a family and children, to consider. Therefore, we controlled for age in years, education in eight levels, and income in SEK hundred thousand. All three variables were taken directly from the Swedish population register.

People may change jobs as a result of other things than exposure to bullying. Feeling uncomfortable or being dissatisfied with one's job may have an influence on one's mental health – something that may change when getting a new job. As the focus of the current study is on the consequences of changing jobs when being bullied, when testing H3, we excluded the Changers who did not self‐label as bullied or who had a score on the NAQ–R of 25 or less. We selected 25 because with that score it is impossible to have reported at least weekly exposure of at least one negative act (i.e., the Leymann criterion for workplace bullying). In other words, such a person will not be categorized as bullied using any of the criteria for bullying previously used in research (cf. Nielsen, Notelaers & Einarsen, 2020; Rosander & Blomberg, 2019).

Often when wanting to predict something at follow‐up, one controls for the dependent variable at baseline. In terms of mental health problems, previous research has clearly shown that there is a strong association between exposure to bullying and mental health problems (see e.g., Nielsen & Einarsen, 2012). In the current study the zero‐order correlations between workplace bullying and mental health problems at baseline were 0.46 and 0.42 for depression and anxiety symptoms respectively. Adjusting for mental health problems at baseline can be done to remove influence of mental health problems at follow‐up unrelated to the actual research interest. However, this would also remove variance connected to what we want to study. The aim of the current study is not to establish an association between bullying at baseline and mental health problems at follow‐up, but merely to investigate the difference in outcome comparing Stayers and Changers. Therefore, we did not adjust for baseline mental health as it would account for the vast majority of variance in the dependent variable, leaving only a fraction as a basis for the current research interest.

There were significant differences between men and women for anxiety symptoms (women having higher scores) at both baseline, *t*(1089) = −4.32, *p* < 0.001, and follow‐up, *t*(1081) = −4.30, *p* < 0.001 – a result that has been shown in previous studies (e.g., Boyd, Van de Velde, Vilagut *et al*., 2015), whereas the results for depression symptoms were practically identical for men and women at both measurement times. To adjust for this difference, we added sex as a covariate for H3b.

### Attrition analyses

To examine the effects of attrition on the overall sample those who completed both questionnaires (“Completers”) were compared to the ones who dropped out after the first round (“Drop‐outs”). We compared a number of demographic variables and also the main study variables. There were no significant differences between Drop‐outs and Completers comparing sex, education, managerial position, or if they had a permanent contract or not. The Drop‐outs were younger (46.6 years, *SD* = 11.8) than the Completers (49.3 years, *SD* = 10.0), *t*(1851) = 5.15, *p* < 0.001. They had also worked a shorter time at the current place of work, 12.3 years (*SD* = 11.5) compared to 13.4 years (*SD* = 11.5) for the Completers. The Drop‐outs had a higher NAQ–R sum and higher score on the HADS, both for anxiety and for depression. For NAQ–R sum the mean score for the Drop‐outs was 28.2 (*SD* = 9.3) and for the Completers was 27.5 (*SD* = 7.2), *t*(1845) = 1.96, *p* = 0.025. For anxiety the Drop‐outs had a mean score of 6.0 (*SD* = 4.2) compared to the Completers who had a score of 5.4 (*SD* = 4.0), *t*(1844) = 2.72, *p* = 0.003. For depression the Drop‐outs has a score of 3.8 (*SD* = 3.5) compared to 3.4 (*SD* = 3.2), *t*(1844) = 2.39, *p* = 0.008.

To further investigate the effects of attrition, the same variables were also used when comparing Drop‐outs, Stayers, and Changers who were bullied at baseline. There were no significant differences between the three groups for sex, education, managerial position or if they had a permanent contract or not. The bullied Drop‐outs were younger, 44.6 years (*SD* = 11.4) than both the bullied Stayers, 48.6 years (*SD* = 9.8) and bullied Changers, 46.0 years (*SD* = 10.6), *F*(2, 306) = 4.76, *p* = 0.009. There were no significant differences for the length of time at the current place of work or income. The bullied Drop‐outs had a higher NAQ–R score, 43.4 (*SD* = 13.8) compared to the bullied Stayers, 40.0 (*SD* = 8.6), and bullied Changers, 40.1 (*SD* = 9.8), *F*(2, 306) = 3.45, *p* = 0.033. The scores on the HADS were almost identical for all three groups.

The attrition analyses showed some differences comparing the Drop‐outs to the overall cohort, however, when comparing those who were bullied at baseline fewer differences were found. The only differences found was that the Drop‐outs were younger and had a somewhat higher NAQ–R score than both the Stayers and the Changers.

### Statistical analyses

All analyses were conducted using IBM SPSS version 26. To test the first hypothesis, we used logistic regression – one analysis using NAQ–R and another for self‐labeled bullying. For the second and third hypotheses we conducted moderation analyses using model 1 in Hayes (2018) PROCESS macro to investigate if the association between independent and dependent variables differs between Stayers and Changers.

### Ethical considerations

The project was approved by the Regional Ethical Review Board at Linköping University, protocol number: 2017/336‐32. Participation was voluntary and the respondents got information about the study so they could give informed consent to participate. The information made clear that the respondents were free to withdraw at any time. To ensure anonymity, we as researchers were never informed about names, addresses or other identifying information – Statistics Sweden handled all these aspects of the study. Once the data collections ended, Statistics Sweden added demographic information to the data taken directly from the Swedish population register and then the data were sent to us.

## RESULTS

Table 2 presents descriptive statistics and intercorrelations for the variables used in the study. Sex (58% women) and Changed jobs (16% changed jobs) are categorical variables.

**Table 2:** Descriptive statistics and intercorrelations for the variables of the study (*n = 1,095*)

	Mean	*SD*	1.	2.	3.	4.	5.	6.	7.	8.
1. Sex	–	–								
2. Age	49.29	10.05	0.00							
3. Education	4.72	1.72	0.16***	−0.16***						
4. Income	4.29	2.07	−0.26***	0.19***	0.26***					
5. Changed jobs	–	–	0.01	−0.18***	0.09**	0.03				
6. NAQ–R (T1)	1.25	0.33	−0.06	−0.12***	−0.04	−0.06*	0.13***			
7. NAQ–R (T2)	1.20	0.30	−0.05	−0.12***	−0.03	−0.01	−0.03	0.60***		
8. HADS D (T2)	0.48	0.44	−0.02	−0.06	−0.04	−0.04	0.02	0.34***	0.41***	
9. HADS A (T2)	0.74	0.55	−0.13***	−0.15***	0.03	−0.10***	0.02	0.30***	0.39***	0.66***

*Note*: Sex (men = 0, women = 1). Education = length of education in eight levels, Income = yearly income in hundred thousand SEK, NAQ–R = Negative Acts Questionnaire–Revised, HADS = Hospital Anxiety and Depression Scale, D = Depression symptoms, A = Anxiety symptoms.

* *p* < 0.05;

** *p* < 0.01;

*** *p* < 0.001.

At baseline 16% (182 employees) were subjected to at least occasional bullying according to the lower cut‐off for the NAQ–R (≥ 33), and 3% (37 employees) were classified as actual victims of bullying (NAQ–R ≥ 45). In total 16% (174 employees) changed jobs between baseline and follow‐up. Of those at least occasionally bullied at baseline (NAQ–R ≥ 33) 28% changed jobs, and of the victims of bullying (NAQ–R ≥ 45) 32% changed jobs. As a comparison, only 14% of the not bullied at baseline changed jobs. In Table 3 we compare the level of bullying for the Stayers and the Changers, and for those bullied at baseline and those not bullied.

**Table 3:** Mean NAQ–R sum for the bullied and not bullied, for those who stayed and those who changed jobs at T1 and T2

	Bullied T1 (NAQ–R ≥ 33)					Not bullied T1 (NAQ–R < 33)				
	NAQ–R sum T1			NAQ–R sum T2			NAQ–R sum T1		NAQ–R sum T2	
	*n*	Mean	*SD*	Mean	*SD*	*n*	Mean	*SD*	Mean	*SD*
Stayers	129	39.98	8.61	35.38	11.32	771	24.89	2.85	25.13	4.13
Changers	51	40.10	9.84	28.88	7.00	123	25.28	2.97	24.87	3.97
*p*		ns		*p* < 0.001			ns		ns	
*Cohen's d*		0.01		0.63			0.14		0.06	

*Note*: NAQ–R = Negative Acts Questionnaire–Revised.

Interesting to note in Table 3 is that for the bullied, there was no difference in the level of bullying at baseline between the Stayers and the Changers, but at follow‐up, that is, when the 28% of the bullied had changed jobs, we saw a clear and significant difference. This suggests that the intensity of bullying does not affect the choice to stay or leave for those exposed to bullying. The drop in NAQ–R sum comparing baseline and follow‐up for this group was significant, *t*(50) = 7.58, *p* < 0.001. At follow‐up the mean for the Changers was even clearly below the cut‐off at 33 on the NAQ–R. We also saw a smaller but significant drop in bullying for the Stayers, *t*(128) = 5.02, *p* < 0.001, which could be attributed to a positive selection at follow‐up. As a comparison with the scores in Table 3, those who were bullied at baseline but did not participate in the follow‐up (*n* = 128) had a mean NAQ–R sum of 43.43 (*SD* = 13.81). This is significantly higher than both the bullied Stayers and Changers, *F*(2, 306) = 3.45, *p* = 0.033.

Testing our first hypothesis, controlling for age, education and income showed that the risk of having changed one's job at follow‐up was 2.5 times greater if bullied at baseline (NAQ–R ≥ 33). When using the other measurement method, self‐labeling as bullied at baseline (at least now and then), the risk odds were almost identical (OR 2.49). The results from the two logistic regression analyses are shown in Tables 4 and 5. The results supported H1.

**Table 4:** Logistic regression analysis prediction changing jobs at follow‐up (exposure to bullying behaviors at baseline)

	OR	95% CI	
Bullying, NAQ–R ≥ 33 (T1)	2.52	[1.71; 3.72]	*p* < 0.001
Age	0.95	[0.94; 0.97]	*p* < 0.001
Education	1.12	[1.01; 1.24]	*p* = 0.037
Income	1.08	[1.00; 1.16]	*p* = 0.059

*Note*: NAQ–R = Negative Acts Questionnaire–Revised; OR = Odds Ratio; CI = Confidence Interval.

**Table 5:** Logistic regression analysis prediction changing jobs at follow‐up (self‐labeled bullying at baseline)

	OR	95% CI	
Self‐labeled bullying (T1)	2.49	[1.33; 4.66]	*p* = 0.004
Age	0.95	[0.94; 0.97]	*p* < 0.001
Education	1.10	[0.99; 1.21]	*p* = 0.077
Income	1.08	[1.00; 1.17]	*p* = 0.044

*Note*: OR = Odds Ratio; CI = Confidence Interval.

**Fig. 1:**
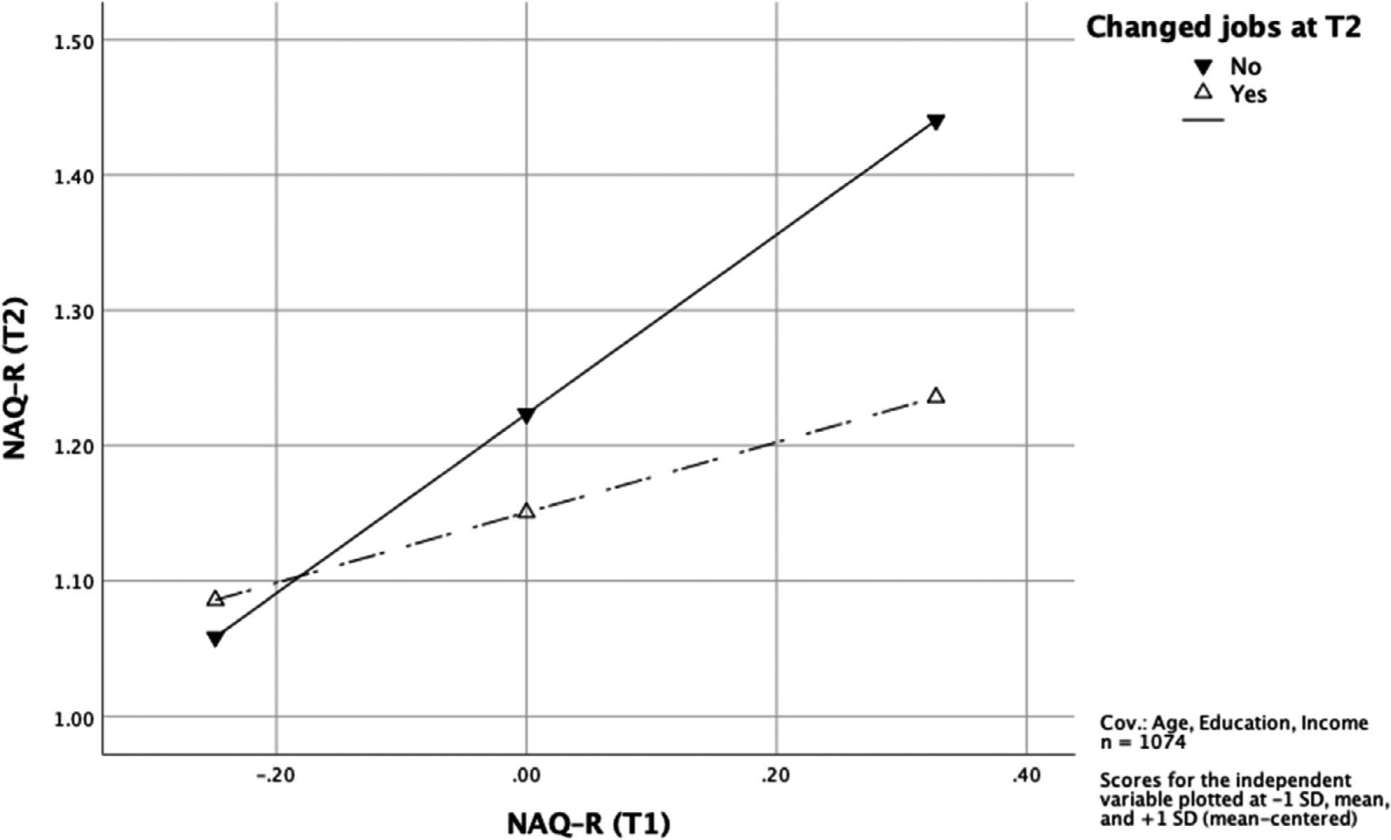
The interaction between changing jobs and workplace bullying at T1 with regard to workplace bullying at T2 (H2).

According to H2, changing jobs should reduce the risk of subsequent bullying. Controlling for the variables that could make it more difficult to change jobs – age, education and income – we tested if having changed jobs moderated the association between workplace bullying at baseline and follow‐up. The results showed a clear interaction, b = −0.40, 95% CI [−0.50; −0.31] as presented in Table 6. The interaction is shown in Fig. 1. Slope tests showed that the associations for both the Stayers, *b* = 0.66, 95% CI [0.61; 0.71], and the Changers, *b* = 0.26, 95% CI [0.18; 0.34], were significant, but with a much larger effect for the Stayers. Thus, the results supported H2.

**Table 6:** Moderation analysis predicting workplace bullying at follow‐up

	*b*	SE b	95% CI	
Bullying, NAQ–R (T1)	1.07	0.07	[0.94; 1.19]	*p* < 0.001
Changing jobs	−0.07	0.02	[−0.11; −0.03]	*p* < 0.001
NAQ–R (T1) x Changing jobs	−0.40	0.05	[−0.50; −0.31]	*p* < 0.001
Age	−0.00	0.00	[−0.00; −0.00]	*p* = 0.004
Education	−0.01	0.00	[−0.01; 0.00]	*p* = 0.234
Income	0.01	0.00	[0.00; 0.01]	*p* = 0.032

*Note*: Dependent variable: NAQ–R (T2). *b* = Unstandardized coefficient; CI = Confidence Interval; Model 1 in the PROCESS macro (Hayes, 2018) was used.

Testing the third hypothesis we did the same type of moderation analysis as for the second hypothesis, but now predicting mental health problems at follow‐up – for depression symptoms (H3a) and for anxiety symptoms (H3b). To reduce the risk of possible influence of people with mental health problems changing jobs for other reasons than exposure to bullying, we excluded those who changed jobs and did not self‐label as bullied and had a NAQ–R sum of 25 or less as described in the methods section.

**Fig. 2:**
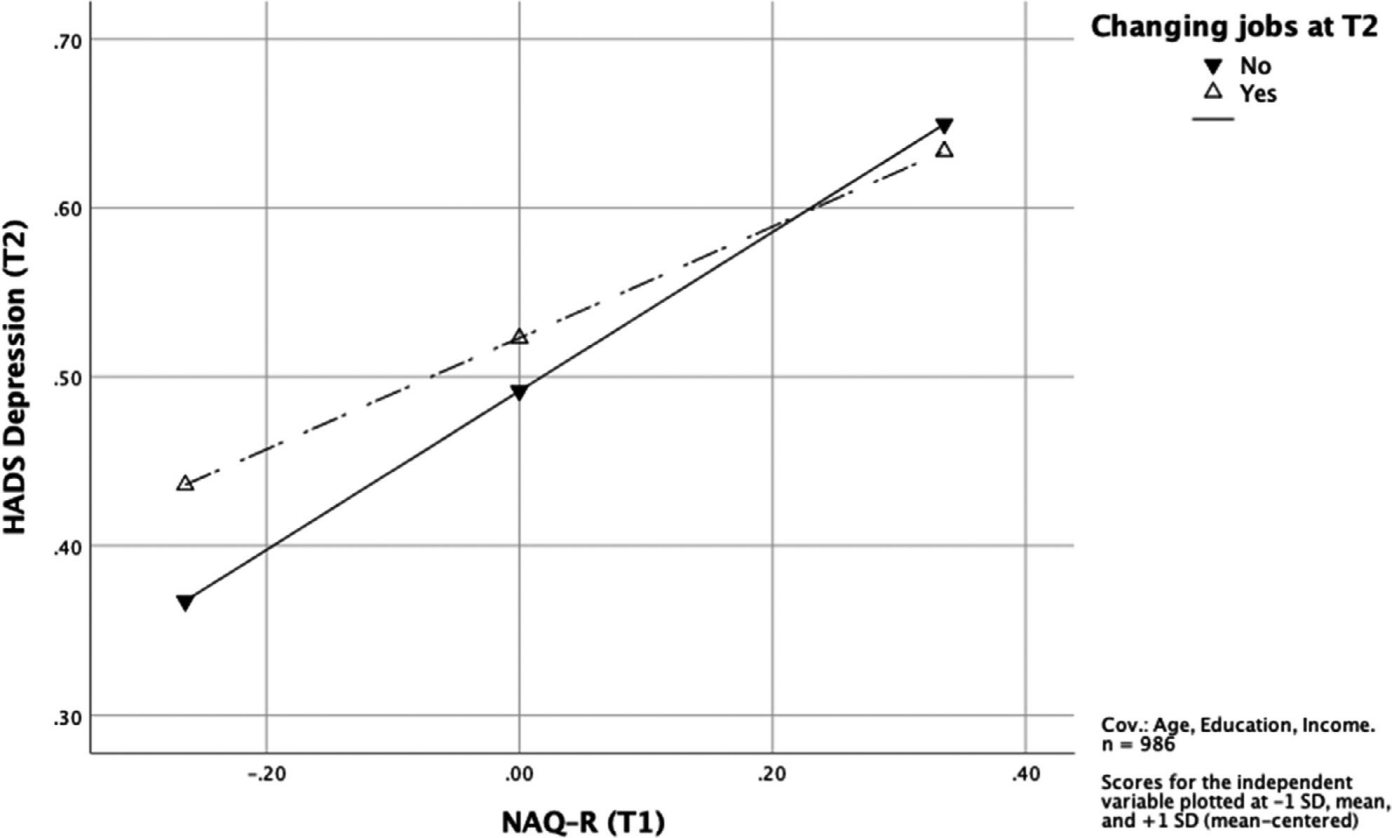
The interaction between changing jobs and workplace bullying at T1 with regard to depression symptoms at T2 (H3a).

**Fig. 3:**
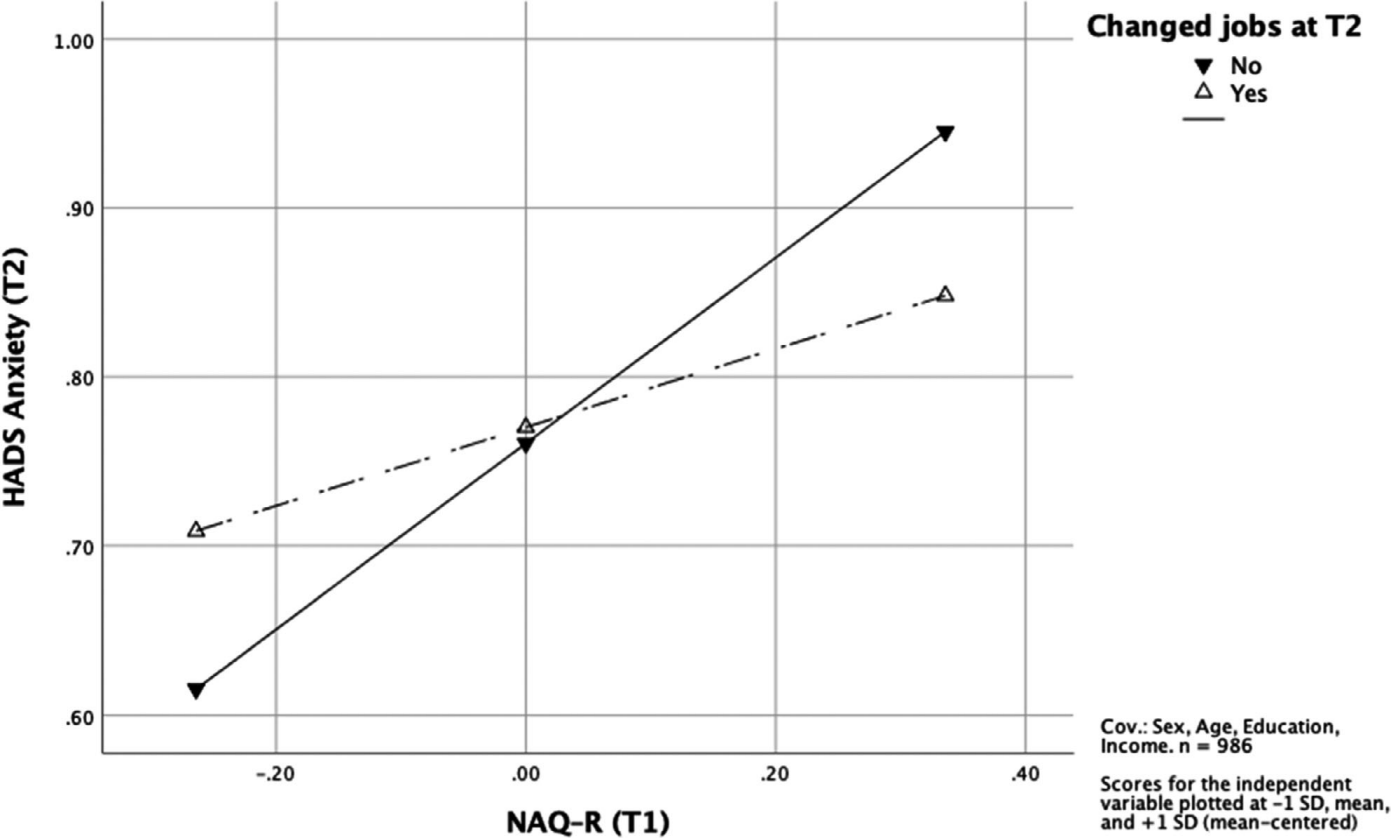
The interaction between changing jobs and workplace bullying at T1 with regard to anxiety symptoms at T2 (H3b).

Controlling for age, education and income, the results showed that changing jobs is not a moderator predicting depression symptoms at follow‐up, *b* = −0.14, 95% CI [−0.36; 0.08] (see Table 7). Figure 2 shows the associations between bullying at baseline and depression symptoms at follow‐up for both Stayers and Changers (no significant interaction). However, changing jobs is a moderator predicting anxiety symptoms at follow‐up (controlling for sex, age, education, and income), *b* = −0.32, 95% CI [−0.59; −0.04] as presented in Table 8. The interaction is shown in Fig. 3. Slope tests showed a positive significant association between workplace bullying at baseline and anxiety symptoms at follow‐up for the Stayers, *b* = 0.55, 95% CI [0.44; 0.66], and a non‐significant association for the Changers, *b* = 0.23, 95% CI [−0.02; 0.48].

**Table 7:** Moderation analysis predicting depression symptoms at follow‐up

	*b*	SE b	95% CI b	
Bullying, NAQ–R (T1)	0.61	0.14	[0.34; 0.88]	*p* < 0.001
Changing jobs	0.03	0.06	[−0.08; 0.14]	*p* = 0.575
NAQ–R (T1) x Changing jobs	−0.14	0.11	[−0.36; 0.08]	*p* = 0.212
Age	−0.00	0.00	[−0.00; 0.00]	*p* = 0.398
Education	−0.01	0.01	[−0.02; 0.01]	*p* = 0.425
Income	0.00	0.01	[−0.01; 0.02]	*p* = 0.815

*Note*: Dependent variable: HADS Depression (T2); *b* = Unstandardized coefficient; CI = Confidence Interval. Model 1 in the PROCESS macro (Hayes, 2018) was used.

**Table 8:** Moderation analysis predicting anxiety symptoms at follow‐up

	*b*	SE b	95% CI b	
Bullying, NAQ–R (T1)	0.87	0.17	[0.53; 1.20]	*p* < 0.001
Changing jobs	0.01	0.07	[−0.13; 0.15]	*p* = 0.891
NAQ–R (T1) x Changing jobs	−0.32	0.14	[−0.59; −0.04]	*p* = 0.023
Sex	0.16	0.04	[0.09; 0.23]	*p* < 0.001
Age	−0.01	0.00	[−0.01; −0.00]	*p* = 0.002
Education	0.01	0.01	[−0.02; 0.03]	*p* = 0.622
Income	−0.01	0.01	[−0.02; 0.01]	*p* = 0.542

*Note*: Dependent variable: HADS Anxiety (T2); b = Unstandardized coefficient; CI = Confidence Interval. Model 1 in the PROCESS macro (Hayes, 2018) was used.

The results supported H3b, showing that changing jobs is a moderator for the association between baseline bullying and anxiety at follow‐up. The association was not significant for the Changers, only for the Stayers. H3a, on the other hand, was not supported, meaning that the association between bullying at baseline and depression symptoms at follow‐up is strong regardless of changing jobs or not, *b* = 0.61; 95% CI [0.34; 0.88]. Changing jobs has an impact on anxiety, but not on depression symptoms, which seem to linger.

To investigate the impact of depression symptoms and the risk of a vicious circle, a follow‐up analysis was conducted to test the reversed association between depression symptoms at baseline and workplace bullying at follow‐up, and if changing jobs is a moderator. Controlling for age, education and income, changing jobs was a moderator, *b* = −0.21, 95% CI [−0.32; −0.09] and a slope test showed that only the association for the Stayers was significant, *b* = 0.26, 95% CI [0.21; 0.30]. For the Changers the association between depression symptoms at baseline and bullying at follow‐up was non‐significant. This points to lingering depression symptoms at follow‐up for the Changers, but the symptoms do not seem have an impact on bullying at the new workplace.

## DISCUSSION

In this study, we investigated the consequences of changing jobs when one is bullied. As a starting point we looked at the risk of changing jobs when bullied. The results showed that those who were subjected to bullying behaviors or self‐labeled as bullied were more likely to change jobs. The risk of actually having changed jobs at follow‐up was about 2.5 times that of those not bullied for both ways of measuring bullying. This is similar to what previous studies have found (Clausen *et al*., 2013; Glambek *et al*., 2015). Extending previous research, we have studied what happens to the level of exposure, but also the consequences of bullying in terms of mental health problems at follow‐up if changing jobs.

When actually changing jobs, we see a dramatic drop in the level of exposure – from a sum score of above 40 to below 29 on the NAQ–R. These are mean scores for all bullied Changers, but the level at follow‐up is clearly below the lower cut‐off for bullying (NAQ–R < 33). We see a drop in the scores also for the Stayers, but it is much smaller. To put the baseline scores in perspective, the ones bullied at baseline who eventually dropped out of the study had a higher score than both the Stayers and the Changers. This could be related to the risk of expulsion from working life faced by victims of bullying. Glambek *et al*. (2015) found a more than fourfold risk of unemployment and a threefold risk of disability retirement over a five‐year period. Based on this it is reasonable to assume that some of the respondents who took part at baseline dropped out because they were no longer part of the workforce.

Overall, it is possible to discern three paths as a consequence of exposure to bullying behaviors: staying at one's current job, changing jobs, or facing expulsion from working life. For the last we do not have any follow‐up data, but for the first two we see a drop in exposure for both groups at follow‐up, although for the Changers the drop is much bigger. It is interesting to note that the variance of the NAQ–R score for the Stayers at follow‐up increased, while for the Changers it decreased. This could indicate a more uncertain situation for the Stayers as a group compared to the Changers. Whereas some Stayers saw a reduction in exposure, possibly as a result of interventions at their place of work, the situation worsened for other Stayers. For Changers the reduction in exposure was more consistent for all.

Workplace bullying is a long‐lasting phenomenon, both by the way it is defined (Einarsen *et al*., 2020) and as evidenced by empirical studies (Zapf *et al*., 2020). This by itself means that the risk of still being exposed at a later point in time is very likely. Previous research suggests that conditions in the work environment are the main cause of bullying – the so‐called work environment hypothesis of workplace bullying (Einarsen, 2000; Leymann, 1996; Salin & Hoel, 2020). We hypothesized that since a change of jobs would also imply a change in work environment, changing jobs would also remove or reduce the risks of exposure to negative behaviors in line with the work environment hypothesis. The results showed that, as hypothesized, the Changers have a weaker association between exposure to bullying behaviors at baseline and follow‐up than the Stayers. There was still a significant association for the Changers, but as discussed above reduction in exposure was significant. There are studies focussing focusing on a vicious circle of bullying in which exposure leads to, for example, mental health problems, which in turn may increase the risk of subsequent exposure (Nielsen *et al*., 2014; Reknes, Pallesen, *et al*., 2014). One explanation for this lies in the possibility of a heightened sensitivity to others' behaviors and of interpreting them as negative. In our study the association between baseline exposure and symptoms of depression at follow‐up was the same for Stayers and Changers. In terms of a vicious circle this could be a risk factor for subsequent bullying at the new workplace. However, as the follow‐up analysis showed, the association between baseline depression symptoms and subsequent bullying was only significant for the Stayers. This could be interpreted as a sign that not only the occurrence of bullying itself is dependent on the work environment, but also that the risks associated with a vicious circle are reduced when changing jobs.

We investigated mental health consequences of workplace bullying and whether changing jobs had an impact. The results showed that the association between bullying at baseline and anxiety at follow‐up was not significant for the Changers – which is in line with the hypothesis. Workplace bullying is an extreme stressor (Zapf & Einarsen, 2005). Moving to a new job at a new workplace, one no longer has to face the bully or bullies from the old workplace. Removing the immediate threat gives the former victim a chance to go back to appraisal processes in relation to the new working environment (Lazarus, 1999), which could result in a normalization of one's relationships and view of the new workplace. This normalization through re‐appraisal could have a direct positive effect on the level of anxiety. However, when victims of bullying are exposed to prolonged negative treatment, their fundamental beliefs in the world are threatened (Janoff‐Bulman, 1989), which could result in extreme emotional reactions and depression. The schema theory of shattered assumptions could help understand that our results showed no difference between the Stayers and the Changers in the association between baseline bullying and subsequent depression. The experience of depression symptoms seems to remain 18 months later if bullied at baseline, no matter if one stays or leaves one's job. A reduction in anxiety, but not in depression was also shown in the study by Brousse *et al*. (2008). When there is a perception of no control and that there is nothing one can do to remedy the situation – helplessness in terms of CATS (Ursin & Eriksen, 2004) – flight, in this case changing jobs, may be a last resort. For many, changing jobs is not an easy decision to make, and for most probably not the first response. Flight as a response to continued arousal is not a response formed from experience and there is no acquired perceived probability of a given outcome (unless one has been bullied in several different contexts and flight has been used successfully before). Flight would represent a final attempt to retain or to take back some control of the situation, but it may also mean throwing oneself into the unknown, hoping for a situation at least not as bad and uncontrollable as the previous situation.

It is interesting to note that the levels of exposure to bullying behaviors at baseline were almost identical for the bullied Stayers and the bullied Changers – a NAQ–R score difference of only 0.12 on a scale from 22 to 110. This shows that the reason for changing jobs was not that the Changers had a higher level of exposure compared to the Stayers and that they too will leave when reaching a higher level of bullying exposure. In terms of Lee and Mitchell's (1994) unfolding model of voluntary turnover, the shock to the system as a result of exposure to bullying behaviors, at which point one starts to think about one's job and if leaving is a reasonable response, could come at any level of exposure. The results do not tell if this is due to more or less being pushed out or if it is more of a survival tactic and an active choice made by the bullied. Previous studies have often framed it such that the bullied is being forced away from the workplace (e.g., Berthelsen, Skogstad, Lau & Einarsen, 2011), and in a way this may be true no matter if the bullied makes an active choice or not. Reasons for leaving are beyond the scope of the current study, but would be interesting to follow up on. The question of what finally tips the scale and makes a bullied employee change jobs would give an insight into the process of bullying and what may alleviate it.

Despite the results we want to raise a word of caution. The remedy for a bad working environment should never be to encourage the ones who are treated badly to leave. In terms of the work environment hypothesis (Einarsen, 2000; Leymann, 1996; Salin & Hoel, 2020), the main reasons for workplace bullying are not to be found on a personal level, but on an organizational level. A bad work environment remains also after those who suffer the most leave, and there is a clear risk that others become new victims. Therefore, it is important to always address the underlying causes. Reviews of organizational risk factors of bullying may provide useful insight how to do this (e.g., Salin & Hoel, 2020). However, on an individual level changing jobs may be helpful. Our results point in that direction. We can show that the level of exposure to bullying behaviors and victimization drops when changing jobs. Also, for those who changed jobs, there is no association between bullying at baseline and anxiety at follow‐up. However, the association to depression symptoms was the same no matter if staying or changing jobs, when bullied at baseline. A practical implication from this is to offer rehabilitation or therapy if possible also to Changers as depression, in a longer perspective, could be a risk factor for future bullying. For example, according to the gloomy perception mechanism put forth by de Lange *et al*. (2005), people with depression may have a more negative outlook on events which could lead to problems in interpersonal relationships at work. These could in turn fuel conflict and be a new starting point for bullying.

### Strengths and limitations

A strength of the current study is that it is based on a probability sample of the whole Swedish workforce with the demographic variables taken directly from the Swedish population register. Measures of bullying and mental health problems were self‐report measures, which could be subjected to biases such as social desirability and common method variance. However, using a time lag of 18 months should have reduced the risk of the latter (Podsakoff, MacKenzie, Lee & Podsakoff, 2003). We know that 174 of the participants had changed jobs in time for the follow‐up. We do not know how many of the people who were bullied at baseline left for unemployment (i.e., were not part of the follow‐up), but it is probable that at least some did, as previous research has shown a great risk for expulsion from working life as a consequence of bullying (Glambek *et al*., 2015). We also do not know when the change of jobs occurred – only that it happened during the 18 months between baseline and follow‐up. There are no reasons to believe anything but that the individual job changes occurred evenly spread over this time period. This means some of the Changers had worked up till a year and a half at the new workplace, while others only had worked a short time. For some, the reduction in exposure to bullying behavior could lie in a “honeymoon phase” at the new workplace, and bullying may commence again at a later stage. Future research needs to follow up what happens to a victim of bullying over time after changing jobs. Also, we do not know if the decision to change jobs was voluntary for all Changers. Exposure to bullying can result in lower ability to fulfill one's tasks and duties, which may be grounds for a termination of the work contract. The law in Sweden prohibits people from being fired because they are bullied – organizations are required to deal with the negative exposure, but in practice it may be hard to prove bullying and it may be easier to see the lack of delivery from an exposed individual. The process and reasoning of the exposed, from the event that jars the employee to the decision to leave, would be interesting to capture, for instance using diary studies. At what point in time in the bullying process does the decision to quit one's job occur? Is it early in the bullying process or late? Are there differences in consequences depending on when it happens? We also do not know if the change of jobs meant a move to a completely new organization or just a change of workplace within the same organization. This is something for future research to study. Should a move within a specific (large) organization suffice to reduce bullying and subsequent anxiety, this may have important implications for HR. However, again we remind that transferring the victim does not in itself solve any of the underlying causes that need to be addressed.

## CONCLUSION

We have shown that changing jobs can be an individual remedy for a victim of bullying, at least in the short run. The level of exposure is reduced significantly if leaving one's current workplace, which gives further support for the work environment hypothesis. As has been shown in previous research and in the current study, employees who are bullied are more likely to change jobs. It is a chance to make a fresh start at a new workplace. Such a change was probably typically experienced as a relief by those exposed to bullying at baseline, as the level of anxiety was significantly reduced for those changing jobs. As for depression, there was no difference between Stayers and Changers. Changing jobs did not reduce the risk of depression if one had been bullied. This points to a need to ensure that victims of bullying get help and support, even if they change jobs. All in all, changing jobs seems to have positive consequences for the individual employees exposed to bullying. However, as discussed above it should only be a last resort. It is important to remember that solving the situation by encouraging the victim to leave will not address any of the underlying causes and shortcomings in the work environment.

## FUNDING

This work was supported by the AFA Insurance under Grant number 160285; the Swedish Research Council for Health, Working life and Welfare under Grant number 2019–01232.

## ETHICAL APPROVAL

The study was reviewed and approved by the Regional Ethical Review Board at Linköping University, protocol number: 2017/336–32.

## AUTHOR CONTRIBUTION

The study conception and initial design was made by Michael Rosander. Denise Salin contributed to the design. Data were collected by Michael Rosander and Stefan Blomberg. Data analysis was performed by Michael Rosander. The first draft of the manuscript was written by Michael Rosander. Denise Salin contributed to the writing of the manuscript. All authors read, commented on, and approved the final manuscript. All the authors contributed to the article and approved the submitted version.

## Data Availability

The data that support the findings of this study are available from the corresponding author upon reasonable request.
